# Regulation of ATG4B Stability by RNF5 Limits Basal Levels of Autophagy and Influences Susceptibility to Bacterial Infection

**DOI:** 10.1371/journal.pgen.1003007

**Published:** 2012-10-18

**Authors:** Ersheng Kuang, Cheryl Y. M. Okumura, Sharon Sheffy-Levin, Tal Varsano, Vincent Chih-Wen Shu, Jianfei Qi, Ingrid R. Niesman, Huei-Jiun Yang, Carlos López-Otín, Wei Yuan Yang, John C. Reed, Limor Broday, Victor Nizet, Ze'ev A. Ronai

**Affiliations:** 1 Signal Transduction and Cell Death Programs, Sanford-Burnham Medical Research Institute, La Jolla, California, United States of America; 2 Institute of Human Virology, Zhongshan School of Medicine, Sun Yat-Sen University, Guangzhou, China; 3 Department of Pediatrics, School of Medicine and Skaggs School of Pharmacy and Pharmaceutical Sciences, University of California San Diego, La Jolla, California, United States of America; 4 Department of Cell and Developmental Biology, Sackler Faculty of Medicine, Tel Aviv University, Tel Aviv, Israel; 5 Department of Anesthesiology, University of California San Diego, La Jolla, California, United States of America; 6 Veterans Administration San Diego Healthcare System, San Diego, California, United States of America; 7 Institute of Biological Chemistry, Academia Sinica, Taipei, Taiwan; 8 Departamento de Bioquímica y Biología Molecular, Universidad de Oviedo, Oviedo, Spain; The University of North Carolina at Chapel Hill, United States of America

## Abstract

Autophagy is the mechanism by which cytoplasmic components and organelles are degraded by the lysosomal machinery in response to diverse stimuli including nutrient deprivation, intracellular pathogens, and multiple forms of cellular stress. Here, we show that the membrane-associated E3 ligase RNF5 regulates basal levels of autophagy by controlling the stability of a select pool of the cysteine protease ATG4B. RNF5 controls the membranal fraction of ATG4B and limits LC3 (ATG8) processing, which is required for phagophore and autophagosome formation. The association of ATG4B with—and regulation of its ubiquitination and stability by—RNF5 is seen primarily under normal growth conditions. Processing of LC3 forms, appearance of LC3-positive puncta, and p62 expression are higher in *RNF5^−/−^* MEF. RNF5 mutant, which retains its E3 ligase activity but does not associate with ATG4B, no longer affects LC3 puncta. Further, increased puncta seen in *RNF5^−/−^* using WT but not LC3 mutant, which bypasses ATG4B processing, substantiates the role of RNF5 in early phases of LC3 processing and autophagy. Similarly, RNF-5 inactivation in *Caenorhabditis elegans* increases the level of LGG-1/LC3::GFP puncta. *RNF5^−/−^* mice are more resistant to group A *Streptococcus* infection, associated with increased autophagosomes and more efficient bacterial clearance by *RNF5^−/−^* macrophages. Collectively, the RNF5-mediated control of membranalATG4B reveals a novel layer in the regulation of LC3 processing and autophagy.

## Author Summary

Autophagy is an intracellular catabolic process by which a cell's own components are degraded through the lysosomal machinery. Autophagy is implicated in various cellular processes such as growth and development, cancer, and inflammation. Using biochemistry, cell biology, and genetic models, we identify a ubiquitin ligase that limits autophagy in the absence of an inducing stimulus (e.g. starvation). The control of basal autophagy is mediated by the ubiquitin ligase RNF5 through its regulation of the membrane-associated ATG4B protease. Using RNF5 mutant mice we demonstrate the implications of this regulation for host defense mechanisms that limit intracellular infection by bacterial pathogens.

## Introduction

Autophagy is an intracellular catabolic process by which cellular components are degraded through the lysosomal machinery. Conserved from yeast to humans, autophagy is fundamental to eukaryotic cell homeostasis [1], [2]. Autophagy functions in diverse cellular processes such as growth and development, cancer, and inflammation [3]–[5], and is implicated in both cell survival and death, depending on the cell type and stress conditions. Accordingly, autophagy has been associated not only with disease progression but also with its prevention [6], [7]. Interestingly, while certain viruses and bacteria can subvert and manipulate autophagic pathways during establishment of infection, autophagy plays a protective role against intracellular replication of several pathogens including group A *Streptococcus* (GAS) [8], [9]. Given the broad importance of autophagy in cell biology, it is of great interest to define the mechanisms underlying its control under normal and stress-related conditions.

Autophagy takes place through a series of steps that include initiation, elongation, and formation of autophagosomes, followed by fusion with lysosomes, and finally maturation and degradation of the autolysosome [10], [11]. Each step in this process involves a number of autophagy (ATG)-specific proteins that control a highly coordinated cascade of events culminating in autolysosome formation [12]. Among these, ATG7 and ATG3 conjugate mammalian LC3 homologues to phosphatidylethanolamine (PE), and ATG7 and ATG10 conjugate ATG12 to ATG5 [13], [14]. The cysteine protease ATG4 contributes to this chain of events by cleaving the LC3 C-terminal domain to generate LC3-I [15]. Consequently, LC3-I is converted by ATG7 and ATG3 to LC3-II, which is essential for phagophore and autophagosome formation [16]–[18]. ATG4 also plays a role in the final step of autophagy by deconjugating LC3-II, enabling LC3 to be released from autolysosomal membranes and recycled [19]–[21].

Four mammalian homologues of yeast ATG4 have been identified: ATG4A, ATG4B, ATG4C, and ATG4D [22]. ATG4B has broad specificity for the mammalian ATG8 homologues GATE-16, GABARAP, and LC3, whereas ATG4C and ATG4D show minimal activities toward LC3 substrates [23], [24]. Following cleavage by caspase, ATG4D stimulates GABARAP-L1 processing and autophagosome formation [25]. Studies of ATG4 gene knockout mice have revealed some differential functions of the isoforms; while *ATG4C^−/−^* mice exhibit marked changes in autophagic activity following prolonged starvation [26], *ATG4B^−/−^* mice show a clear reduction of basal- and starvation-induced autophagy in all tissues, associated with impaired proteolytic cleavage of LC3 orthologs [27].

The availability of LC3 is regulated co-translationally, suggesting that ATG4B is not a limiting factor in the control of LC3 processing and the early stages of autophagy [16]. Nonetheless, accumulative evidence suggests that ATG4B is regulated in a manner that has concomitant effects on LC3 processing. For example, upregulating ATG4 by Egr1 or ARH1 is associated with increased LC3 processing and autophagy in lung tissues and ovarian cancer [28], [29]. Moreover, disruption of ATG4B inhibits processing of LC3 paralogues and autophagy [27], [30], [31]. Collectively, these observations identify a critical role for ATG4B in control of autophagy. While growing evidence suggests that LC3 processing is induced prior to the formation of the pre-initiation ATG1/13 complex, the mechanisms controlling basal and induced levels of LC3 are largely unknown. Here, we have investigated the relationship between ATG4B activity and LC3 processing, and demonstrate that the ubiquitin ligase RNF5 controls the stability, and hence availability, of a membrane-localized fraction of ATG4B. Accordingly, RNF5 limits ATG4B processing of membranal LC3 with concomitant effects on autophagy.

RNF5 (also named RMA1), an 18-kDa RING finger E3 ligase, has been implicated in *C. elegans* muscle structure through its control of UNC-95, a LIM domain-containing protein involved in maintenance of dense bodies, the muscle attachment sites [32] and in the molting process [33]. In mammalian cells, RNF5 regulates cell motility by ubiquitinating the focal adhesion protein paxillin [34]. Deregulated expression of RNF5 in the muscle of RNF5 transgenic mice results in the formation of inclusion body myositis, an endoplasmic reticulum (ER) stress-associated muscular disorder [35]. A link to ER stress was also demonstrated through RNF5's role in ER-associated protein degradation (ERAD), where it contributes to clearance of misfolded proteins [36], [37]. RNF5 promotes tumor cell resistance to cytoskeletal-targeting anticancer agents, and its expression is inversely correlated with survival in breast cancer and melanoma patients [38]. Lastly, RNF5 contributes to the cellular response to infection by controlling the stability of the mitochondrial proteins MITA and MAVS, which are involved in antiviral innate immune signaling [39], [40]. RNF5-dependent ubiquitination of SOP-A affects *Salmonella* trafficking from endosomes/vacuoles to the cytosol [41].

Given the multiple effects of RNF5 on ER stress, innate immunity, and bacterial infection, processes that are each influenced by autophagy, we have examined the possibility that RNF5 may play a role in the control of autophagy. In the present study, we demonstrate that RNF5 negatively regulates basal levels of autophagy by mediating the ubiquitination and degradation of a membranal-associated pool of ATG4B. Macrophages from RNF5 knockout (KO) mice exhibit more efficient processing of GAS, and RNF5 KO mice are less susceptible to lethal infectious challenge by this leading bacterial pathogen.

## Results

### RNF5 interacts with and ubiquitinates ATG4B

**Figure 1:**
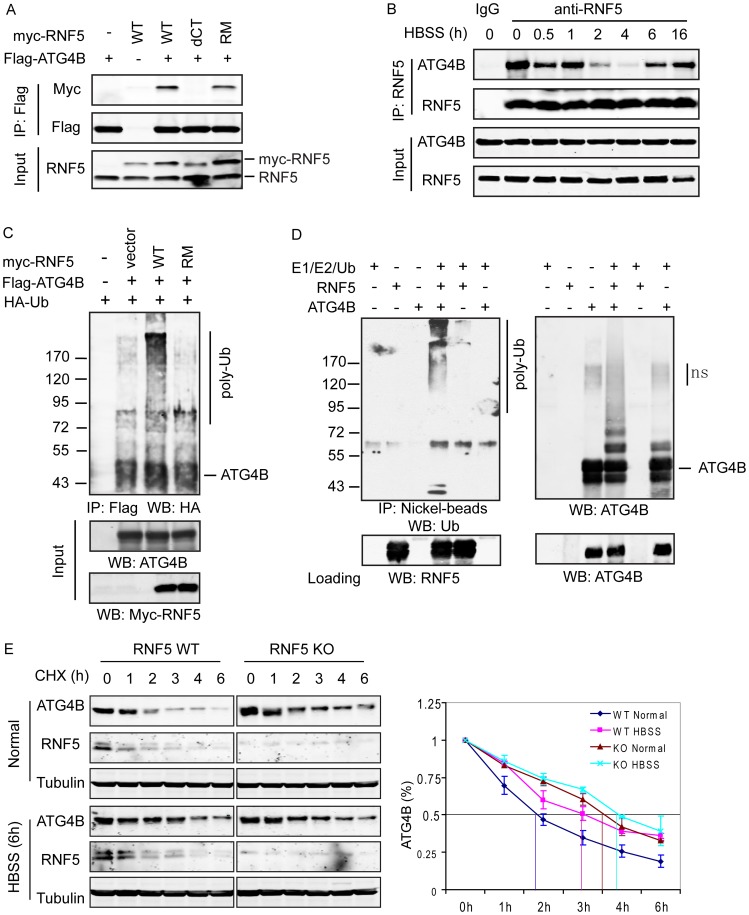
RNF5 interacts with ATG4B and mediates its ubiquitination and degradation. (A) Membrane-bound RNF5 interacts with ATG4B. RNF5 WT, C-terminal TM-deleted mutant (dCT), and RING domain mutant (RM) constructs were co-transfected with Flag-ATG4B. The cell lysates were immunoprecipitated with anti-Flag antibodies conjugated to beads, then immunoblotted with the indicated antibodies. (B) Dynamic interaction of ATG4B and RNF5 during starvation-induced autophagy. The interaction between endogenous ATG4B and RNF5 expressed in HeLa cells was monitored at the indicated time points prior to and following HBSS-induced starvation. Cell lysates were immunoprecipitated and immunoblotted with the indicated antibodies. (C) In vivo ATG4B ubiquitination by RNF5. HeLa cells were co-transfected with Flag-ATG4B, HA-ubiquitin (Ub), and WT or RM RNF5 plasmids. After 24 h, cells were treated with MG132 (10 µM) for 4 h and lysed with buffer containing 1% SDS. The lysates were immunoprecipitated with anti-Flag antibody in the presence of 0.1% SDS, followed by immunoblotting with anti-HA antibodies. (D) ATG4B in vitro ubiquitination by RNF5. Purified His6-tagged ATG4B was bound to nickel beads and incubated for 1 h at 37°C with RNF5 in the presence of the indicated in vitro ubiquitination reagents. The bead were washed three times with PBS containing 0.1% SDS and 0.2% Triton X-100 and then immunoblotted with antibodies to ATG4B and Ub. (E) RNF5 reduces ATG4B stability. Half-life of ATG4B in RNF5 WT and KO MEFs under normal growth conditions or after HBSS treatment was determined by addition of cycloheximide (CHX) (40 µg/ml) for the indicated times. Cell extracts were subjected to immunoblot analysis using anti-ATG4B and anti-tubulin antibodies. Quantitation of ATG4B levels based on band intensity was measured using the LICOR system, and is shown as the mean of duplicate experiments.

Given the various effects of RNF5 on ER stress and innate immune pathways, processes that are influenced by autophagy, we examined the possibility that RNF5 may play a direct role in the control of autophagy. A cDNA library screen using a yeast-based functional assay for ATG4B inhibitors identified RNF5 as a candidate regulator (1 in 12 hits among 2×10^5^ colonies; Figure S1). To confirm a possible interaction of RNF5 with ATG4B, we examined the association between exogenous and endogenously expressed proteins. Immunoprecipitation of exogenously expressed WT or activity-dead mutant (C74A) forms of ATG4B confirmed their association with exogenously expressed RNF5 (Figure S2a). ATG4B also associated with the RING mutant (RM) form of RNF5, indicating that the ubiquitin ligase activity of RNF5 was not required for this interaction. However, RNF5 lacking its membrane-spanning C-terminal domain (ΔCT) was no longer able to interact with ATG4B (Figure 1A), suggesting that the interaction takes place within the membranal domain. The latter is consistent with earlier studies showing that RNF5 interactions with, and effects upon, its substrates require membrane anchoring [36].

Given the role of ATG4B in autophagy, we monitored the RNF5–ATG4B interaction following exposure of cells to Hank's balanced salt solution (HBSS), a commonly used method to induce starvation with concomitant autophagy. Surprisingly, the interaction between ectopically expressed RNF5 and ATG4B in 293T cells was transiently reduced within 2–4 h following HBSS treatment, then returned to initial levels as early as 6 h after treatment (Figure S2B). This pattern was confirmed in HeLa cells, where the interaction between endogenous RNF5 and ATG4B was present prior to HBSS treatment, decreased within 2–4 h of autophagy induction then resumed between 6–16 h post-treatment (Figure 1B). These observations suggest that an interaction between RNF5 and ATG4B exists under basal conditions, but is attenuated following exposure of cells to autophagy stimuli. This implies that RNF5 may have a negative regulatory role to limit autophagy under normal growth conditions.

RNF5 and ATG4B contain multiple cysteines that have been implicated in their respective activities; Cys26 and Cys30 in RNF5 RING domain, among a total of seven cysteines, and Cys74 and Cys78 within the catalytic domain of ATG4B, among a total of 12 cysteines [32], [34], [42]. Therefore, we assessed whether reducing conditions could affect the interaction between RNF5 and ATG4B. Treatment of cells with the thiol-reducing agents dithiothreitol (DTT), glutathione (GSH) or H_2_O_2_ attenuated the ATG4B–RNF5 interaction (Figure S2C). These observations suggest that reducing conditions affects RNF5 interaction with ATG4B, as one mechanism underlying RNF5 association with and control of ATG4B stability, consistent with the reported role of reducing agents and ROS on the control of ATG4B and autophagy [42].

To determine whether the RNF5–ATG4B interaction has implications for ATG4B processing of LC3, we manipulated RNF5 expression in 293T cells and then assessed the interaction between ATG4B and LC3. Inhibition of RNF5 expression with established shRNA [35], [38] increased the level of ATG4B–LC3 interaction, while conversely RNF5 overexpression reduced the interaction, compared to cells transfected with control vectors (Figure S2D). These data indicate that the RNF5–ATG4B interaction affects ATG4b association with LC3, which is expected to affect LC3 processing.

Among the different ATG4 members, ATG4B was found to exhibit the highest affinity for RNF5 (Figure S3A). Among other members of the ATG family, including ATG3, ATG5, and ATG7, only ATG4B exhibited an appreciable association with RNF5 (Figure S3B). Consistent with these observations, there was no significant change in the levels of ATG3, ATG5, or ATG7 in MEFs prepared from RNF5-deficient animals (Figure S3C). Mapping RNF5–ATG4B interaction domains identified both N- and C-terminal domains (amino acids 61–126 and 320–393) of ATG4B (Figure S3D) and C-terminal domain of RNF5 (aa121–180; Figure S3E) as those required for the interaction between these two proteins.

### RNF5 induces ATG4B ubiquitination and proteasome-mediated degradation

Having identified the interaction between RNF5 and ATG4B, we next examined whether this interaction affects ATG4B stability, given the E3 ubiquitin ligase activity of RNF5. To determine if RNF5 ubiquitinates ATG4B constructs for the two proteins were co-expressed with HA-tagged ubiquitin in HeLa cells. ATG4B ubiquitination was induced in the presence of the WT but not the RING mutant form of RNF5 (Figure 1C). To confirm that ATG4B was directly ubiquitinated by RNF5, we performed in vitro ATG4B ubiquitination in the presence of RNF5. Notably, poly-ubiquitinated ATG4B was observed in the presence of E1 and E2 enzymes, ubiquitin, and RNF5 (Figure 1D). These results suggest that RNF5 directly induces ATG4B ubiquitination.

We next assessed whether RNF5-mediated ubiquitination affects ATG4B stability. We initially assessed the steady-state levels of ATG4B in cells in which RNF5 expression was increased or inhibited. The steady-state level of ATG4B was higher in *RNF5^−/−^* MEFs compared to WT cells (Figure S2D). The half-life of ATG4B was measured in cycloheximide (CHX) chase experiments comparing RNF5 KO to WT MEFs. Consistent with results obtained under steady-state conditions, the half-life of ATG4B increased from 1.75 to 3.5 h in RNF5 KO cells that were maintained under normal growth conditions. Following exposure to HBSS, the half-life of ATG4B was prolonged further in the absence of RNF5, albeit to a lesser degree (from 3 h to just over 4 h) (Figure 1E), consistent with the pattern of RNF5–ATG4B association (Figure 1B, Figure S2B). Similarly, shRNA inhibition of RNF5 expression in WT MEFs efficiently prolonged ATG4B half-life, whereas overexpression of RNF5 reduced ATG4B stability (data not shown). In agreement, reduced ATG4B protein, seen upon ectopic expression of RNF5, was attenuated in the presence of the proteasome inhibitor MG132 (Figure S4A). Notably, addition of MG132 increased the level of ATG4B expression in both RNF5 WT and KO MEF cells, although the degree of increase was more pronounced in the WT cells (Figure S4B). The latter suggests that other ubiquitin ligases may also contribute to the regulation of ATG4B stability. Consistent with these observations, a similar degree of ATG4B ubiquitination was seen in MG132 treated WT and RNF5 KO cells, although without MG132 WT cells exhibited higher degree of ATG4B ubiquitination compared with the RNF5 KO cells (Figure S4C). These results establish that RNF5 regulates ATG4B stability through its ubiquitination and proteasome-dependent degradation.

### RNF5 regulation of ATG4B affects LC3 processing

Having shown that RNF5 regulates ATG4B stability, and having obtained initial evidence that this affects ATG4B–LC3 interactions, we next explored the implications of these observations on ATG4B activity and autophagy. An established fluorogenic assay that measures ATG4B cleavage of an in vitro-synthesized pro-LC3 substrate was employed [43]. Using immunopurified ATG4B proteins prepared from control or shRNF5-expressing cells, we found that the in vitro activity of ATG4B was 40–60% higher in cells with reduced RNF5 expression (Figure S5A). To investigate the role of RNF5 in LC3 processing, we detected intracellular proteolysis of LC3 based on non-conventional secretion of Gaussia luciferase. In this system, LC3 is anchored in the cell by fusion to β-actin and the cleaved product is secreted. Thus, ATG4B activity can be monitored by quantifying extracellular luciferase activity resulting from the proteolytic cleavage of the LC3 site between Gaussia luciferase and β-actin [44], [45]. Corroborating our in vitro findings, intracellular proteolysis of LC3 measured by the luciferase assay increased in shRNF5-expressing HeLa cells, and conversely, was attenuated following ectopic expression of RNF5 (Figure S5B). These results indicate that RNF5 expression regulates ATG4B activity and LC3 cleavage.

**Figure 2:**
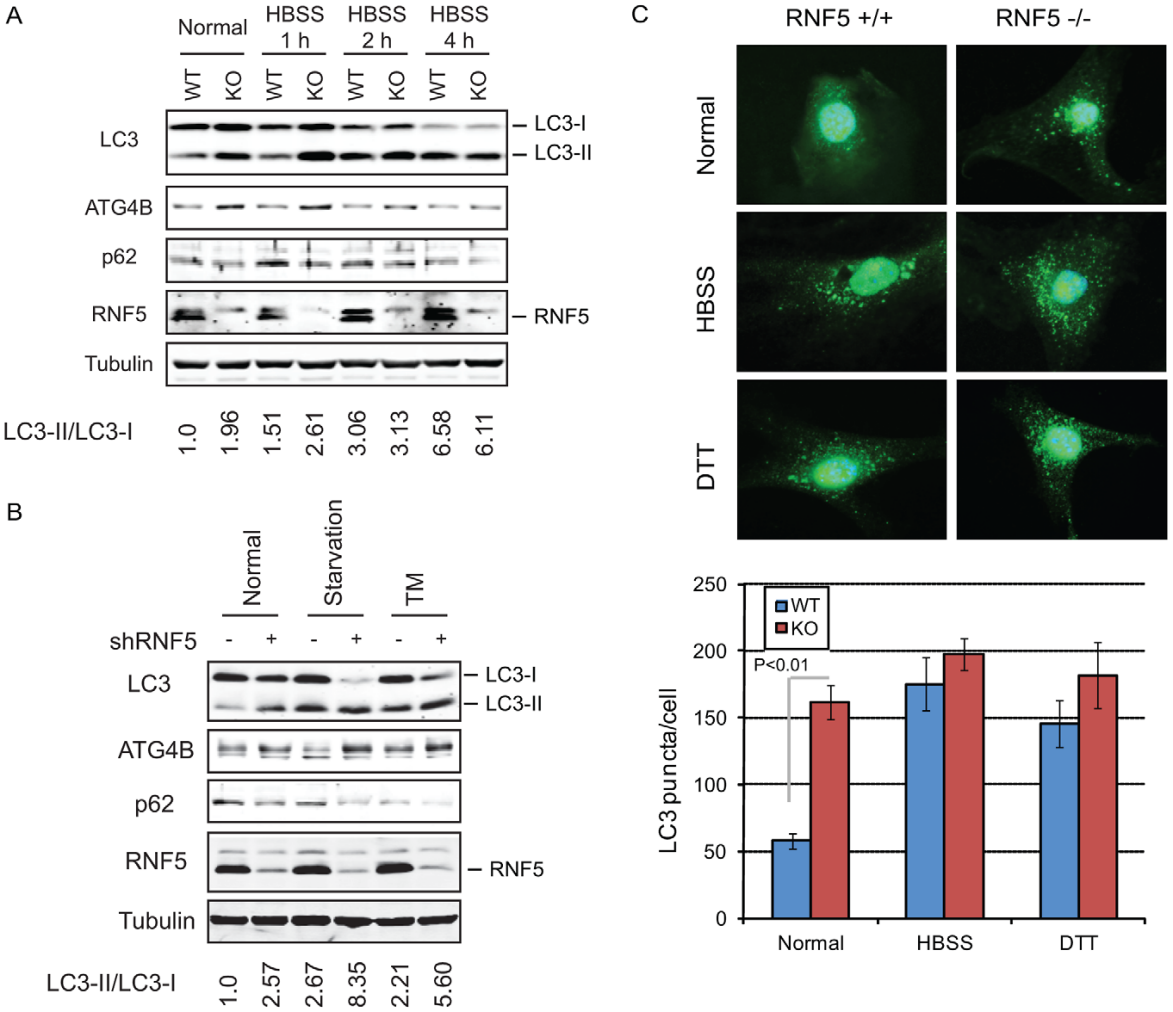
RNF5 negatively regulates autophagy. (A) Immunoblot analysis of LC3 and p62. RNF5 WT and KO MEFs were maintained in HBSS for 1 to 4 h before cells were lysed, and proteins were resolved and analyzed by immunoblotting with the indicated antibodies. (B) Scrambled and shRNF5-transduced PC3 cells were maintained in normal medium, serum-starved overnight, or treated with tunicamycin (TM, 5 µg/ml, 8 h). (C) Amount of endogenous LC3 puncta is affected by RNF5. RNF5 WT and KO MEFs grown in normal medium, starved with HBSS (2 h), or treated with DTT (5 mM, 8 h) were fixed and immunostained with anti-LC3 antibody. Results show the quantification of endogenous LC3 puncta counted in >20 cells per experimental condition, in duplicate.

To pursue this finding further, we monitored the effect of RNF5 on the appearance of different intracellular LC3 forms by western blotting. We monitored changes in the processing of LC3 in WT and RNF5 KO MEFs prior to, or 1, 2, and 4 h after treatment with HBSS (Figure 2A). Loss of RNF5 expression enriched in expression of the matured LC3 form, LC3-II, with the greatest difference being observed at the earlier time points (Figure 2A; control and 1 h, compared with 2 or 4 h). The latter is consistent with the finding that RNF5–ATG4B association is seen prior and at early time points following autophagy stimuli (Figure 1B). Neither GABARAP nor NBR1 expression were altered under the same conditions (Figure S6A). Notably, following LC3-II or LC3 decoration at later time points (6 and 8 h) did not reveal changes (not shown), probably since RNF5 affects to lesser degree the course of autophagy, once triggered. Inhibition of RNF5 led to the appearance of the processed LC3-II form in cells growing under normal conditions, and has increased the relative amount of LC3-II forms in cells that were subjected to starvation or after induction of ER stress with tunicamycin (Figure 2B). These data demonstrate that disruption of RNF5 expression affects LC3 processing under normal growth conditions, while also contributing to Atg4B availability following induction of autophagy. The level of LC3-II in both WT and RNF5 KO cells was elevated following treatment with the lysosomal inhibitors E64A and pepstatin A (Figure S6B). Similarly, degradation of the autophagy marker p62 was elevated upon inhibition or depletion of RNF5 expression (Figure 2A, 2B). Collectively, these data point to a role for RNF5 as a negative regulator of autophagy.

### RNF5 affects autophagosome formation

The appearance of LC3-II has been associated with autophagosome formation, which can be visualized by the appearance of LC3-positive puncta [15], [17]. We therefore monitored the effect of RNF5 on the accumulation of endogenous LC3-positive puncta. Compared with WT cells, RNF5*^−^* ^/*−*^ MEFs maintained under normal growth conditions contained higher levels of LC3-positive puncta, consistent with the appearance of the LC3-II form. This difference was not apparent in cells subjected to starvation or ER stress stimuli (Figure 2C). These results substantiate the notion that RNF5 negatively regulates ATG4B availability with concomitant effect on LC3 processing and autophagy under normal growth conditions. Correspondingly, inhibition of RNF5 leads to increased basal levels of autophagy.

**Figure 3:**
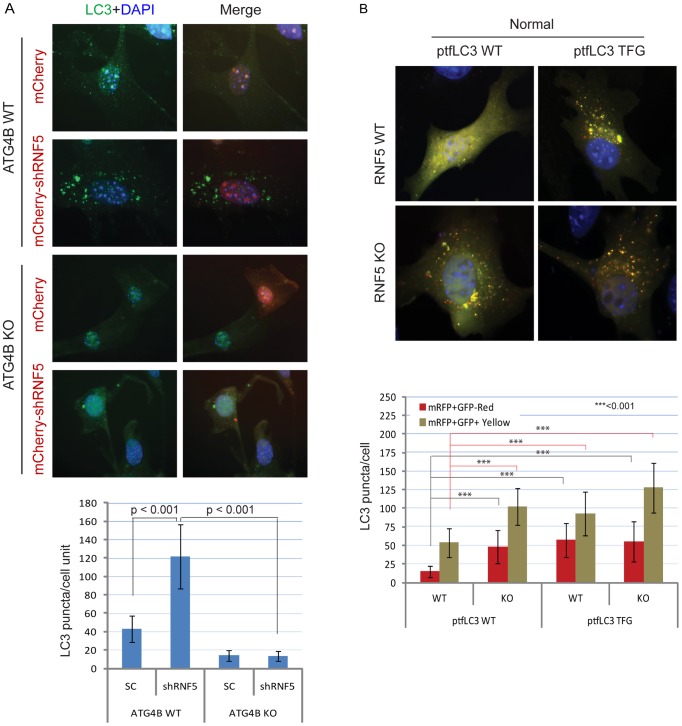
RNF5 regulates autophagosome formation. (A) ATG4B is required for RNF5-mediated endogenous LC3 puncta. ATG4B WT and KO MEFs were transfected with mCherry harboring RNF5 or control shRNA, and 48 h later cells were fixed and immunostained with anti-LC3 antibody. Shown are the mean values for LC3-positive puncta in >20 cells per experimental condition. SC, scrambled shRNA. The right column depicts merged LC3 (green) and RNF5 (mCherry) and DAPI channels. (B) Immunofluorescence images of mRFP-GFP-LC3 in RNF5 WT and KO MEFs. The plasmid ptfLC3 expressing the LC3 WT or LC3 mutant containing a pre-cleaved LC3 TFG were transfected into RNF5 WT and KO MEFs. After 24 h, the cells were fixed and the number of LC3-positive red or yellow puncta was counted in >20 cells. Quantification was confirmed by an independent analyst who was blinded to the sample identities.

To determine whether this change in LC3 processing can be associated with the effect of RNF5 on ATG4B, we monitored changes in LC3 puncta in WT or *ATG4B^−/−^* MEFs in the presence of shRNF5. As shown in Figure 3A, inhibition of RNF5 expression increased the number of LC3 puncta (three times) in WT, but not in *ATG4B^−/−^* cells. Support for ATG4B-dependent effect of RNF5 on LC3 processing comes from the use of RNF5 that is truncated in its C-terminal transmembrane domain (ΔCT), a mutant that retains its E3 ligase activity, but no longer interacts with ATG4B (Figure 1A, Figure S3C). Ectopic expression of RNF5 ΔCT (Figure S6C, S6D) in *RNF5^−/−^* MEF did not reduce the number of LC3 puncta, as seen with the WT RNF5 (Figure S6C). These findings substantiate that the negative regulation of LC3 puncta by RNF5 occurs via its regulation of ATG4B.

We next assessed whether processing of LC3 by ATG4B can promote autophagosome formation and maturation in the absence of RNF5 expression. We monitored the formation of autophagosomes and autolysosomes using the mRFP-GFP-LC3 construct [46]. Notably, we also tested a corresponding mRFP-GFP-LC3 mutant in which the C-terminal TFG residue has been exposed such that its sequence mimics pre-cleaved LC3-I, and thus is no longer subject to cleavage by ATG4B [15]. Because GFP is more sensitive than RFP to the acidic environment of lysosomes, the tandem RFP-GFP-LC3 protein will label autophagosomes as yellow (GFP/RFP) puncta, and autolysosomes as red (RFP) puncta. Thus, quantification of yellow and red puncta allows the determination of the effect on autophagosomes versus autolysosomes, respectively. In ATG4B WT-expressing cells, the LC3 TFG construct contained 2-fold more LC3 puncta (both yellow and red) than the WT LC3 construct, indicating that pro-LC3 cleavage by ATG4B promotes the formation of autophagosomes and autolysosomes (Figure S7A). As expected, cells containing the WT LC3 construct formed lower number of puncta in *ATG4B^−/−^* cells than in ATG4B WT cells (Figure S7A). However, the pre-cleaved LC3 TFG mutants still formed, albeit 2-fold fewer, puncta in the *ATG4B^−/−^* cells compared to WT cells (Figure S7A), suggesting ATG4B contributes to additional steps in the autophagy process.

Expression of WT LC3 led to higher levels of both yellow and red puncta in *RNF5^−/−^* versus WT cells under normal (Figure 3B), but not starvation (Figure S7B) conditions, suggesting that absence of RNF5 promotes the formation of both autophagosomes and autolysosomes under basal conditions. In clear contrast, WT and *RNF5^−/−^* cells expressing the pre-cleaved LC3 mutant formed similar numbers of red LC3 puncta (autolysosome), consistent with ATG4B-dependent conversion of pro-LC3 to LC3-I. Taken together, these results suggest that disruption of RNF5 results in elevated autophagosome formation through upregulation of ATG4B, which in turn increases LC3 processing to promote formation and maturation of autophagosomes.

### RNF5 controlsLC3/LGG-1punctain *C. elegans*

**Figure 4:**
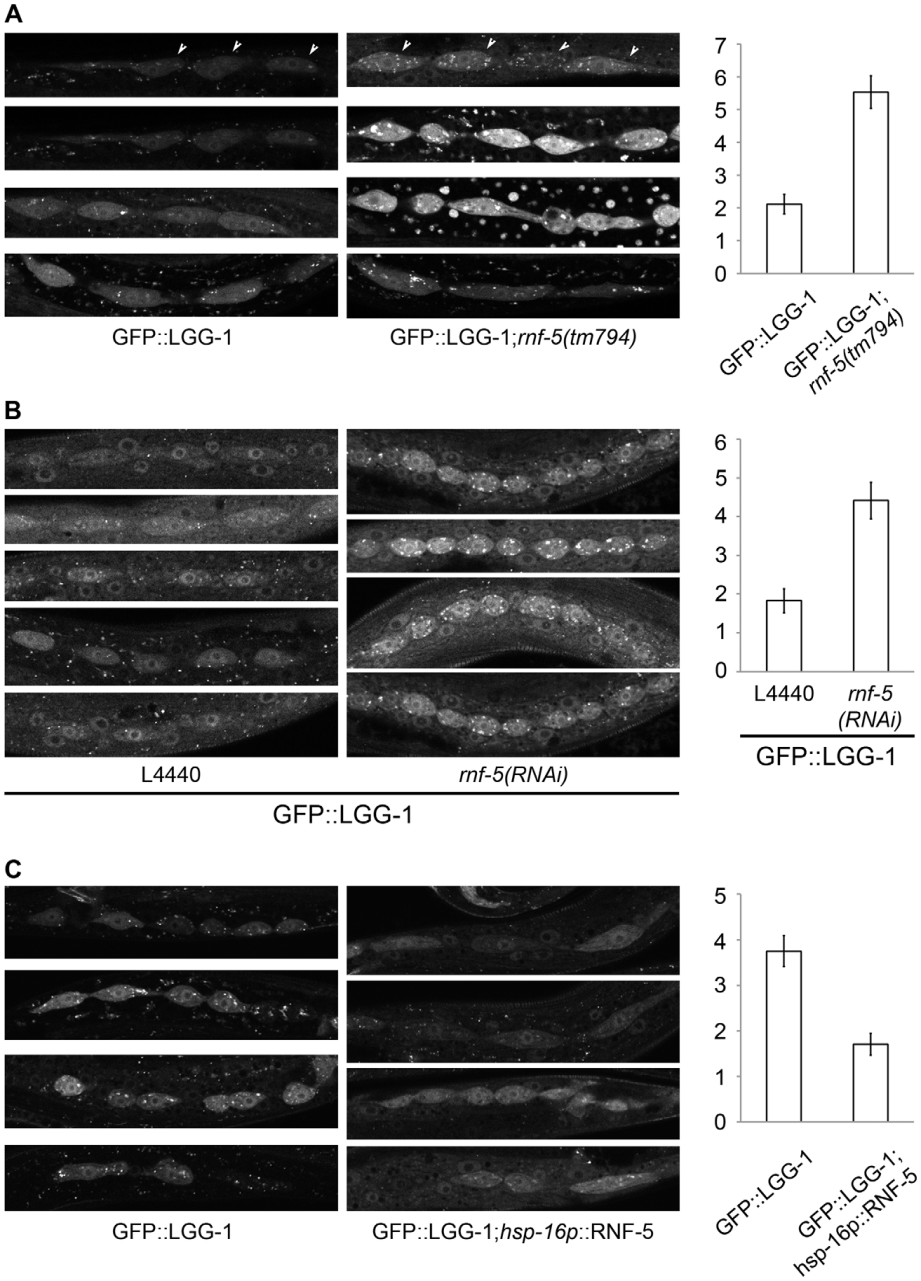
RNF-5 regulates autophagy in *C. elegans*. (A–C) Representative images of seam cells from L3 larvae expressing the GFP::LGG-1 transgene, and the average number of GFP::LGG-1 puncta in seam cells. Three to four independent experiments were performed for each condition. Error bars are ± SEM. P value was calculated using an unpaired two-tailed t-test. (A) *rnf-5(tm794)* larvae had an average of 5.53±0.49 puncta/cell compared to 2.11±0.30 puncta/cell in WT (36 cells from 9 *rnf-5(tm794)* larvae, and 46 cells from 10 WT larvae, p<0.0001). (B) *rnf-5(RNAi)*-treated worms had an average of 4.42±0.47 puncta/cell compared to 1.83±0.31 puncta/cell in control animals (77 cells from 15 *rnf-5(RNAi)* larvae and 64 cells from 17 control larvae, p<0.0001). (C) Animals grown constantly at 25°C: *hsp-16p*::*rnf-5* larvae had an average of 1.71±0.24 puncta/cell compared to 3.75±0.34 puncta/cell in the non-transgenic population (69 cells from 18 *hsp-16p*::*rnf-5* larvae, and 68 cells from 15 non-transgenic larvae, p<0.0001).

We next asked if RNF5 also regulates autophagy in *C. elegans.* To accomplish this, we used *rnf-5(tm794)* deletion mutant that encodes a 36 amino-acid protein lacking the RNF5 RING domain, and thus predicted to be inactive [34]. *rnf-5(tm794)* L3 larvae expressing the autophagy marker GFP::LGG-1 had 2.6-fold more puncta in their seam cells compared to larvae on the WT background (Figure 4A, p<0.0001). This increase in GFP::LGG-1 puncta was also detected in worms treated with *rnf-5(RNAi)* (Figure 4B, 2.4-fold, p<0.0001). Conversely, animals expressing RNF-5 under the heat shock promoter [34] that were grown at 25°C (Figure 4C, conditions that increase the number of puncta in WT worms) resulted in decreased number of GFP::LGG-1 puncta in the seam cells of animals with elevated RNF-5 levels compared to the control animals at 25°C (2.2-fold; p<0.0001). These data suggest that RNF5 is a negative regulator of autophagy in *C. elegans*. Notably, degree of ER stress, measured via the *hsp-4::gfp* transcriptional reporter [47], confirmed that depletion of *rnf-5*does not exhibit increased *hsp-4* transcription (Figure S8).

### RNF5 regulates ATG4B at the membranal compartment

**Figure 5:**
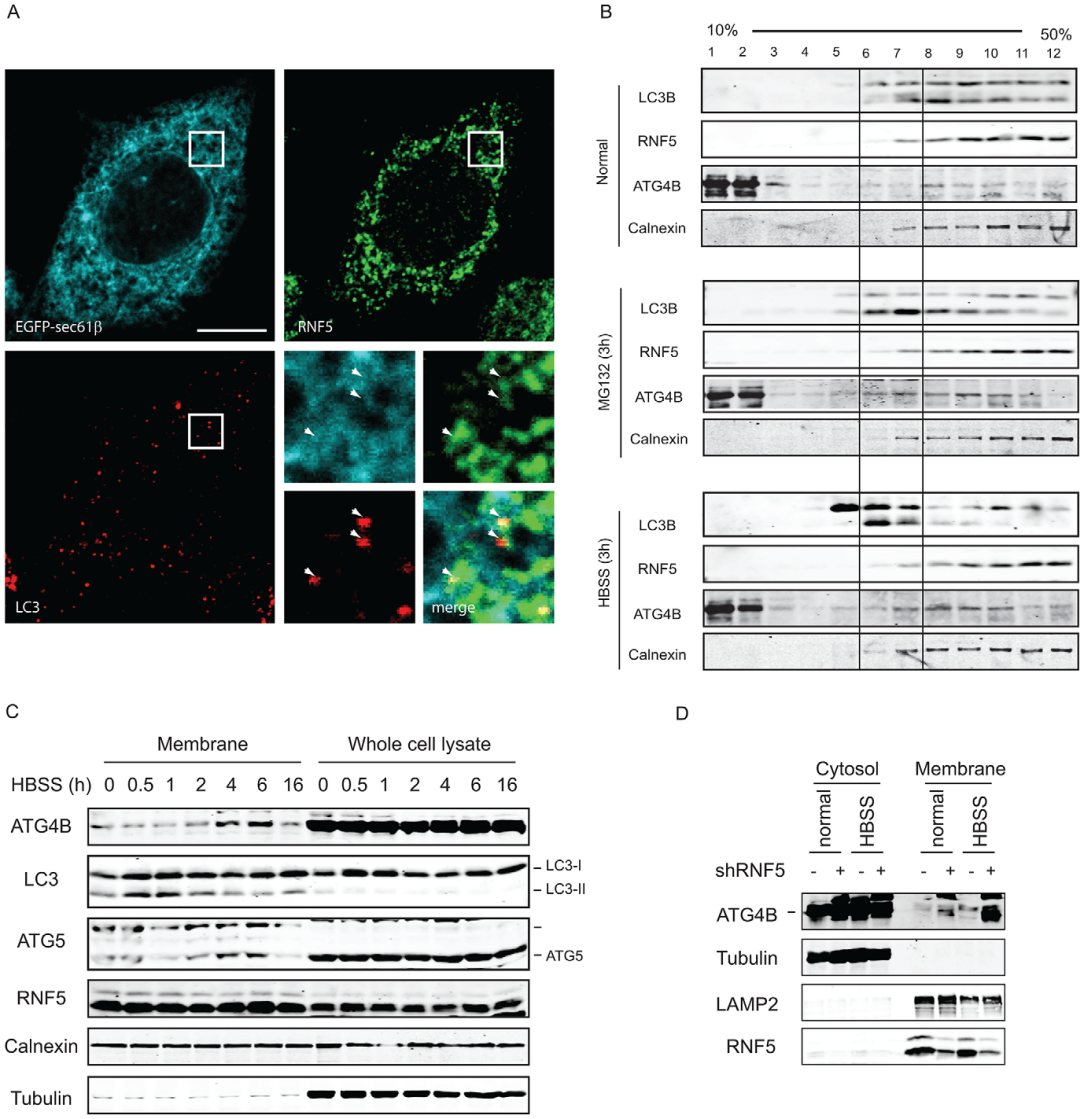
RNF5 co-localizes with—and limits—ATG4B at the membrane compartment. (A) RNF5/LC3 immunofluorescence images in HeLa cells expressing GFP-sec61b. Magnified view of the white square region is shown on the bottom right panels; white arrows denote LC3 foci that colocalized with ER-localized RNF5. Scale bar, 10 microns. (B) ATG4B and RNF5 co-fractionate in ER and autophagosomes. 293T cells were maintained under normal medium or HBSS starvation (3 h) were collected and passed through a 25-gauge needle 10 times and the membrane fractions were enriched and loaded to 10–50% linear sucrose gradient and subjected to ultracentrifugation (160,000 g for 2.5 h). Twelve fractions (1 ml each) were collected from the top to the bottom, and analyzed by western blots as indicated. Calnexin, ER membrane marker. (C) 293T cells were starved using media supplemented with HBSS, and the cells were collected at the indicated time points. Membrane fractions were separated by sucrose gradient ultracentrifugation. Proteins (40 µg/lane) were resolved and immunoblotted with the indicated antibodies. Top, ER-associated GFP-LC3 puncta; Middle, cytosolic GFP-LC3 puncta. (D) PC3 cells stably expressing shRNF5 were maintained in HBSS (3 h) or regular medium. Membrane fractions were separated by sucrose gradient ultracentrifugation. Proteins (40 µg/lane) were resolved and immunoblotted with the indicated antibodies.

Because RNF5 is a membrane-bound E3 ubiquitin ligase, and since it requires its membrane-anchor to affect ATG4B and autophagy we assessed whether RNF5 activity towards ATG4B may preferentially take place at membranal domains. The autophagy markers LC3 and the ER membrane marker Sec61 were used to determine degree of RNF5-colocalization with LC3 at the membranal fraction. Analysis was performed in both MEFs and HeLa cells grown under normal conditions. In HeLa cells we found that 74% of the LC3/RNF5 colocalized spots were within the sec61b structures, whereas in the MEFs this percentage was found to be 83% (Figure 5A). This data suggest that RNF5 effect on LC3 processing does take place within the ER domain.

Biochemical analysis of subcellular fractions, enabling enrichment of membrane domains confirmed that under normal growth conditions RNF5 colocalizes with the ER marker calnexin (Figure 5B), but not with ATG9 (not shown), a marker for the trans-Golgi network and late endosomes, which is redistributed in phagophores/autophagosomes under starvation conditions [48]. Both LC3-I and LC3-II co-fractionated with RNF5 and calnexin under normal growth conditions (Figure 5B, upper panel fractions 7–12). Following HBSS treatment, level of ATG4B increases within the membranal fractions, and LC3B forms are found at earlier fractions, compared with normal growth conditions (Figure 5B, lower panel, fractions 5–7). These results demonstrate that RNF5 colocalizes with components of autophagic machinery within the membranal fractions. To further assess whether RNF5 regulates ATG4B at membranal domains we monitored membrane fractions for the presence of ATG4B during autophagy. Notably, ATG4B and ATG5 were found in the membrane-enriched fractions and their abundance in these fractions increased 2–6 h after starvation (Figure 5C). This pattern of ATG4B localization is consistent with the finding that RNF5 dissociates from ATG4B 2–4 h after HBSS treatment (Figure 1B). ATG4B levels within membrane-enriched fractions were increased in cells that expressed RNF5-targeted shRNA or that were subjected to HBSS treatment (Figure 5D). Collectively, these observations suggest that RNF5 regulates basal autophagy *via* control of ATG4B at the membranal ER compartment.

### Susceptibility to bacterial infection in RNF5 KO mice

**Figure 6:**
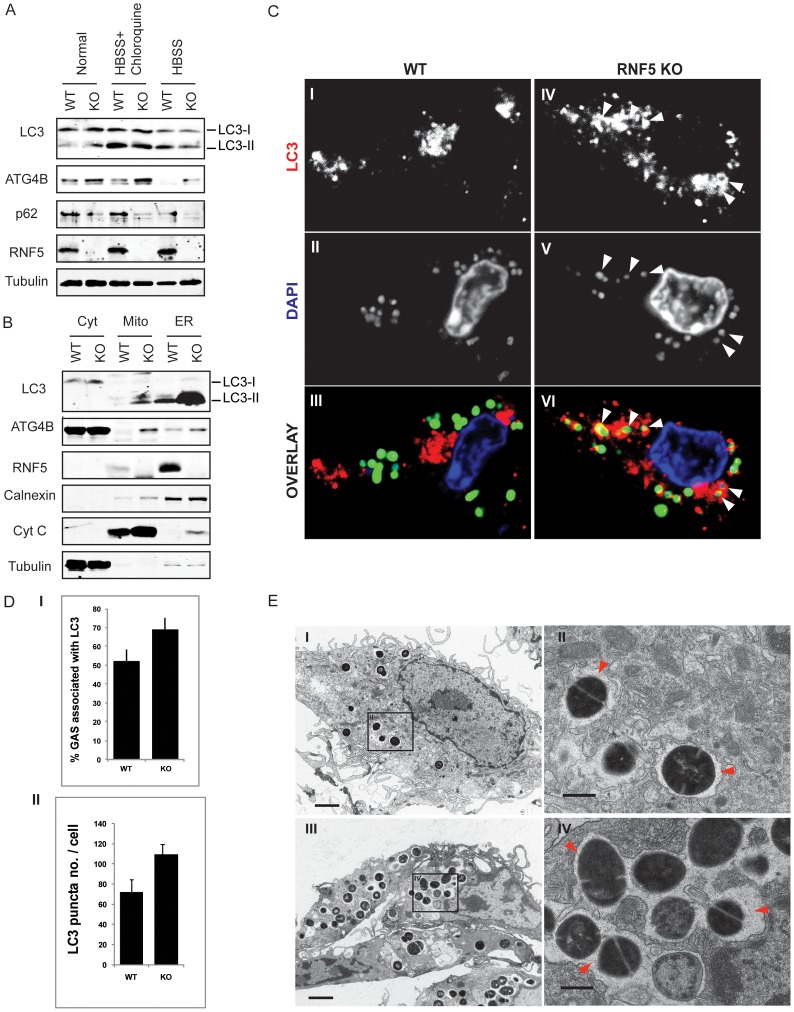
RNF5 affects engulfment of GAS in autophagosomes. (A) Immunoblotting analysis of LC3 and p62 in RNF5 WT and KO macrophages maintained in normal or HBSS-containing media. (B) Subcellular localization of LC3 and ATG4B in macrophages of RNF5 WT and KO mice. Cell extracts were prepared from bone marrow-derived macrophages and fractionated using sucrose gradient ultracentrifugation. Fractions (40 µg protein/lane) enriched for endoplasmic reticulum (ER), mitochondria (Mito), or cytosolic (Cyt) were resolved and analyzed by western blotting with the indicated antibodies. Markers representing each of the organelles are shown. (C) Confocal microscopic images of GFP-labeled GAS (green) and immunostained LC3 (red) depicts representative images of GAS contained in LC3-positive compartments (arrowheads) in bone marrow-derived macrophages from WT and RNF5 KO mice (DNA labeling by DAPI in blue). Bone marrow-derived macrophages were seeded on glass coverslips (5×10^4^/well in 24-well plates) and the following day infected with GAS (MOI = 10) for 2.5 h before being washed and fixed in 3% PFA. Cells were processed for immunofluorescence microscopy using affinity-purified rabbit anti-LC3 IgG, followed by goat anti-rabbit Alexa-488 F(ab′)2, and DAPI to stain the bacterial and cellular DNA. (D) Quantification of GAS associated with LC3-positive autophagosomes in macrophages of WT and RNF5 KO mice. RNF5 KO macrophages contain more GAS engulfed by LC3-decorated structures per cell (I, n = 20, P = 0.077) and more LC3 puncta per cell (II, n = 20, P<0.05). Graphs show the mean ± SE, P value was calculated by Student t-test. Images were acquired on an Olympus FluoView 1000 confocal microscope using a 100× oil immersion lens. (E) EM-based ultrastructural analysis of GAS-infected macrophages from WT (I) and RNF5 KO (II) mice. Within 2.5 h after macrophage infection, most GAS are engulfed within autophagosome-like vacuoles (arrowheads). Scale bar = 2 µm (I).

Given the demonstration that RNF5 controls basal autophagy activity, and the fact that autophagy levels are associated with altered susceptibility to intracellular bacterial replication [8], we next asked if RNF5 WT and KO mice exhibit differences in their response to bacterial challenge. In these studies, we employed the leading human pathogen GAS, previously identified to be susceptible to autophagy-mediated intracellular clearance [9]. Bone marrow-derived macrophages isolated from *RNF5^−/−^* mice demonstrated increased levels of LC3, ATG4B, and decreased level of p62 compared to *RNF5^+/+^* macrophages (Figure 6A), suggesting they had a higher basal level of autophagy. Furthermore, levels of ATG4B and LC3 were elevated in mitochondrial and ER-enriched fractions of *RNF^−/−^* macrophages compared to *RNF5^+/+^* macrophages (Figure 6B). These observations are consistent with membrane anchoring of RNF5 and suggest that RNF5 limits ATG4B-dependent LC3 processing in both mitochondrial and ER membranes. Autophagy-deficient (*ATG5^−/−^*) cells have been shown to be more susceptible to GAS intracellular proliferation [9]. In agreement with those findings, we found higher numbers of LC3 puncta in in *RNF5^−/−^* macrophages infected with GAS than *RNF5^+/+^* macrophages (Figure 6C, 6D), pointing to a more efficient bacterial processing by autophagy in the absence of RNF5. Ultrastructural analysis of GAS-infected macrophages using EM confirmed that higher numbers of bacteria were engulfed in *RNF5^−/−^* macrophages than in WT cells (Figure 6E).

**Figure 7:**
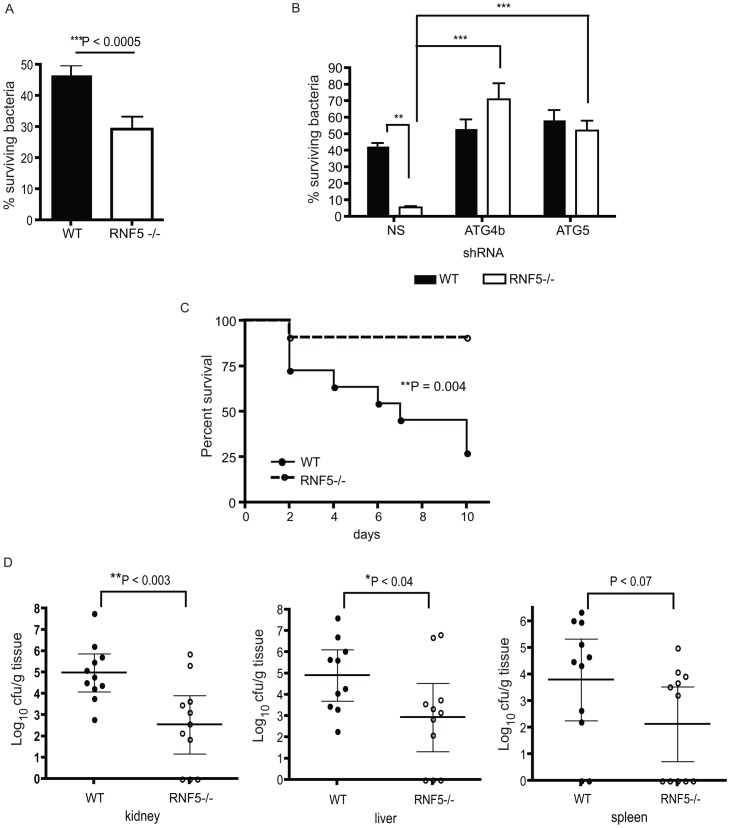
Resistance to GAS infection in RNF5 KO mice. (A) Intracellular killing of GAS is enhanced in *RNF5^−/−^* bone marrow-derived macrophages compared to WT macrophages. Data represent three independent experiments. Differences between groups were analyzed using an unpaired Student's t-test with two-tailed P values. (B) Enhanced killing of GAS by *RNF5^−/−^* macrophages is abolished by knockdown of ATG4B. Bone marrow-derived macrophages were transduced with lentivirus-based nonsense (NS), ATG4B, or ATG5 shRNA and intracellular killing of bacteria was subsequently analyzed. One representative of three independent experiments is shown and differences between groups were analyzed using one-way ANOVA with the Tukey-Kramer post-test. **P<0.01, ***P<0.001. (C) Survival of WT and *RNF5^−/−^* mice (n = 11 for each group from two individual experiments) was monitored over 10 days after intraperitoneal infection with 2×10^7^ cfu of GAS. Comparison of survival curves was performed with the log-rank (Mantel-Cox) test. (D) Bacterial load in the kidney, liver, and spleen of WT and *RNF5^−/−^* mice (n = 11 for each group from two individual experiments), 3 days post-infection with GAS. Data are shown with mean and 95% confidence intervals, and differences between groups were analyzed using an unpaired Student's t-test with two-tailed P values.

GAS intracellular survival was significantly reduced in *RNF5^−/−^* macrophages compared to WT controls (Figure 7A). However, the enhanced intracellular bacterial clearance by *RNF5^−/−^* macrophages was abolished when cells were transfected with ATG4B shRNA (Figure 7B). These results specifically link the enhanced bacterial clearance in the *RNF5^−/−^* macrophages to increased basal autophagy in these cells. Using a murine model of systemic GAS infection, at a challenge dose that produced 75% mortality in WT mice, almost all *RNF5^−/−^* mice survived (Figure 7C). The bacterial loads in mouse organs during the early stages of infection were higher in *RNF5^+/+^* mice compared to *RNF5^−/−^* mice (Figure 7D). Taken together, our data show that the negative regulation of autophagy by RNF5 influences the outcome of GAS infection, and demonstrate for the first time a protective role for autophagy in an in vivo model of this important human infectious disease.

## Discussion

Our findings demonstrate that the ubiquitin ligase RNF5 functions to limit basal levels of autophagy by regulating ATG4B stability. Our conclusion is supported by two genetic models—*C. elegans* and mice—in which inactivation of RNF5 resulted in higher levels of autophagosomes decorated by LC3 puncta. These observations were substantiated by studies with cultured cells in which manipulation of RNF5 altered ATG4B expression and concomitantly affected LC3-II formation and association with autophagosomes. Notably, control of ATG4B by RNF5 primarily occurs prior to and following autophagy, as reflected in the decreased ATG4B–RNF5 interaction between 2 and 6 h after stimulation of autophagy. Correspondingly, effects of RNF5 on ATG4B and autophagy were seen, albeit to lesser degree, following autophagy stimulation. These data indicate that RNF5 limits basal levels of autophagy in the absence of stimulatory factors. Significantly, the regulation of ATG4B by RNF5 primarily takes place at the membranal domains, thereby restricting the effect to the relatively small pool of ATG4B that reaches the membrane domain. This finding suggests that formation of phagophores from cellular membranes may be primarily regulated by a membranal pool of ATG4B, through the membrane-anchored E3 ligase RNF5. Thus, the current study describes a previously unknown aspect of ATG4B regulation, through its availability for autophagosome formation, and thus for autophagy. Current dogma suggests that cleavage of pro-LC3 by ATG4B is regulated co-translationally, and thus, LC3-I is not the limiting factor in the early stages of autophagy. Our findings uncover an important regulatory role for a fraction of ATG4B that must be located at membrane domains and is important for modification of LC3 and subsequent phagophore formation. The importance of RNF5 in this process is supported by the use of mRFP-GFP-LC3 construct containing a truncated ATG4B recognition sequence, circumventing the need to be modified by ATG4B. This construct was no longer subject to regulation by RNF5, demonstrating a pivotal role for RNF5 in ATG4B function. Consistent with this finding, LC3 localization within autophagosomes and autolysosomes was inversely correlated with the level of RNF5 expression. LC3 colocalized with RNF5 within the ER membrane, unless autophagy or proteasomes were inhibited, enabling colocalization.

The implications of this newly discovered layer in the control of autophagy are reflected in the decreased susceptibility of RNF5-deficient mice to bacterial infection with GAS. The higher basal levels of autophagy in these mice allow more efficient clearance of invading bacteria, reducing both the load and physiological consequences of infection, as reflected in the improved survival of *RNF5^−/−^* mice following infectious challenge.

Our findings raise the question as to which physiological cues might regulate the RNF5–ATG4B association/dissociation. It is likely they will involve post-translational modifications of one or both binding partners. Initial data supports the role of ROS in the association between RNF5 and ATG4B (both proteins contain cysteine-rich domains that are required for their function), which is consistent with a reported role for ROS in autophagy [42]. As a membrane-anchored protein, RNF5 must retain its membrane localization to associate with and affect the function of ATG4B, consistent with our earlier findings for other RNF5 substrates [32], [35], [36]. While the extreme carboxyl terminal domain of RNF5 contains its membrane-spanning region and is required for its anchor to the membrane [32], [35], the central to the carboxyl terminal domains was found to be required for its association with ATG4B. Control of ATG4B by RNF5 is likely limited to the small pool of ATG4B localized at these membranes, consistent with phagophore formation from cellular membranes [49], [50]. However, based on the experiments using the LC3-GFP-RFP our data also point to a role for ATG4B in autophagy, other than its role in LC3 processing. This is in agreement with the emerging concept that certain stimuli can elicit autophagy independently of ATG4B activity [51].

Our findings also add to the existing links between the ubiquitin–proteasome system and autophagy. First, the ubiquitin ligase Parkin is linked to mitophagy as well as autophagy, primarily through its function in mitochondrial membrane organization [52]. However, Parkin substrates that directly contribute to autophagic activity have yet to be identified. Second, both p62 and Nbr1 contain ubiquitin-binding domains (UBAs), which are implicated in recruitment of ubiquitin chain-conjugated misfolded proteins to autophagic vesicles [53]. Third, thapsigargin, a model compound often used to induce ER stress, was recently shown to control autophagosome–lysosome fusion, a critical step in the late phases of autophagy, thus revealing an additional link between ER stress and autophagy [54]. Given the number of ubiquitin ligases involved in the control of the ER stress response in general, and ERAD in particular, one would expect that some of these ligases would also affect distinct phases of the autophagy process.

It is clear there is a growing link between autophagy and the clearance of invading bacteria. Initially, genetic inactivation of essential *Atg* genes in *C. elegans* and *Dictyostelium* was shown to alter the fate of invading bacteria [55]. In vitro studies have also shown that inactivation of autophagy enhances *Salmonella* replication in macrophages [56]. Consistent with these studies, we found that macrophages from *RNF5^−/−^* mice contain a greater number of autophagosomes surrounding bacterial pathogens than do cells from WT mice. Of note, it has previously been shown that mice defective in intestinal expression of the *Atg16L1* or *Atg5* genes have defects in gut secretion of antimicrobial peptides [57]. Thus, in addition to restricting the multiplication of intracellular bacteria, future studies could explore whether RNF5 affects the production and secretion of antimicrobial peptides to influence intestinal microbial colonization

In conclusion, our data establishes the presence of a new layer in the control of autophagy through limiting basal levels of autophagy. This control is mediated by the ubiquitin ligase RNF5 through its regulation of the membranal ATG4B protease. We further demonstrate the implications of this regulation for host defense mechanisms that limit intracellular infection by bacterial pathogens, suggesting the possible development of RNF5 inhibitors as new means for treatment of select pathogens and possibly means to overcome their commonly experienced resistance.

## Materials and Methods

### Ethics statement

All animal work has been conducted according to relevant national and international guidelines in accordance with recommendations of the Weatherall report and approved by the IACUC committee at SBMRI and UCSD.

### Cell lines and mice

HeLa cells and PC3 cells were cultured in RPMI 1640 medium with 10% heat-inactivated fetal bovine serum (FBS) and antibiotics. 293T cells were cultured in Dulbecco's modified Eagle's medium supplemented with 10% FBS and antibiotics. RNF5 WT and KO MEFs were prepared from 13.5-day mice embryos following the standard protocol, and were cultured in Dulbecco's modified Eagle's medium supplemented with 10% FBS and antibiotics. RNF5 KO and WT mice were described previously [35] and bred and maintained by the animal facility in our institute (AUF08-008). ATG4B WT and KO MEFs were a gift from Dr. Carlos López-Otín (Universidad de Oviedo, Oviedo, Spain).

### Plasmids and siRNA

The plasmids expressing Myc-RNF5, Flag-RNF5, Flag-RING domain mutant (RM), Flag-C-terminal transmembrane domain-deleted mutant (dCT), and shRNF5 have been described previously [36], [38]. The reporters of intracellular proteolysis based on non-conventional secretion of Gaussia luciferase Actin-DN and Actin-LC3-DN were a gift from Dr. Brian Seed (Massachusetts General Hospital, Boston, MA). Flag-ATG4B and ATG4BC74A were previously reported [43]. Tandem mRFP-GFP-LC3–expressing plasmid ptfLC3 was purchased from Addgene. mRFP-GFP-LC3 TFG was generated by inserting a stop-codon after the C-terminal TFG residue using Quikchange mutagenesis. Lentivirus-based shRNAs of ATG4B and ATG5 were purchased from Sigma validated MISSION shRNA, and prepared following the standard procedure.

### Antibodies and chemicals

Rabbit polyclonal anti-ATG4B, -ATG5, -ATG7, -LC3B, and anti-Flag M2 antibodies were purchased from Sigma. Rabbit anti-LC3 antibody was purchased from MBL. Anti-Ub, anti-p62, anti-GFP, anti-HA, anti-myc antibodies, and protein A/G affinity gels were purchased from Santa Cruz Biotechnology. Tunicamycin were purchased from Calbiochem. The anti-RNF5 antibody was previously described [35], [38].

### Immunoprecipitation and Western blotting

Briefly, cells were lysed with lysis buffer (50 mM Tris-HCl, pH 7.4, 150 mM NaCl, 1% NP-40, 10% glycerol, 40 mM β-glycerophosphate, 30 mM sodium fluoride, 5 mM EDTA, 1× protease inhibitor cocktail (Roche), and 1 mM phenylmethylsulfonyl fluoride). For immunoprecipitation, the cell lysates were incubated with antibody for 1 h and then incubated with protein A/G affinity gels for 4 h or overnight at 4°C. After washing three times with lysis buffer, the protein-bead complexes were resolved by SDS-PAGE (20 µg protein/lane), transferred to nitrocellulose membranes, and subjected to western blotting. The membranes were blocked with 5% dried milk in phosphate-buffered saline (PBS) plus 0.2% Tween 20 and then incubated with primary antibodies for 2 h at room temperature or overnight at 4°C. Anti-rabbit, anti-goat, or anti-mouse IgG antibodies conjugated to horseradish peroxidase (Pierce) or fluorescent dyes (Invitrogen) were used as the secondary antibodies. Blots were visualized by direct imaging on a Licor Odyssey or with an enhanced chemiluminescence system (Pierce).

### In vitro and in vivo ubiquitination

In vitro and in vivo ubiquitination were described previously [36]. Briefly, for in vitro ubiquitination, His6-tagged ATG4B protein was expressed in *E. coli* strain BL21 and purified using nickel-agarose beads. The bead-bound ATG4B was resuspended in buffer (50 mM Tris-HCl, pH 8.0, 5 mM MgCl_2_, 2 mM ATP, 0.5 mM DTT, 2 mM NaF) prior to addition of ubiquitin and ubiquitin system enzymes. The final concentrations were as follows: 200 ng/µL ubiquitin, 2 ng/µL E1, 20 ng/µL E2, and 200 ng/µL substrate in a total volume of 25 µL. The reactions were incubated for 60 min at 37°C, then bead-bound material was washed three times with washing buffer (50 mM Tris-HCl, pH 8.0, 150 mM NaCl, 0.2% Triton X-100, 0.1% SDS) prior to analysis by western blotting. For in vivo ubiquitination, cells were transfected with the indicated plasmids together with a plasmid expressing HA-tagged ubiquitin. Harvested cells were lysed in 2% SDS in TBS (10 mM Tris-HCl, pH 8.0) at 95°C for 10 min. The lysates were diluted 20-fold with TBS containing 0.2% Triton X-100 and 2 mM EDTA to give a final SDS concentration of 0.1%, then incubated on a shaker at 4°C for 30 min. The lysates were incubated for 30 min at 4°C with protein A/G beads and clarified by centrifugation for 10 min (14,000 rpm) at 4°C. For immunoprecipitation, the lysate was incubated with anti-Flag antibody at 4°C for 1 h before protein A/G beads were added and incubated for a further 2 h. Beads were washed three times with TBS containing 0.2% Triton X-100 and 0.1% SDS. Proteins were resolved by SDS-PAGE, transferred, and immunoblotted with the indicated antibodies.

### Immunofluorescent staining

Cells were fixed with 4% formaldehyde in PBS for 15 min, permeabilized with 0.2% Triton X-100 in PBS for 10 min, and blocked with 2% bovine serum albumin in PBS for 30 min. Cells were then incubated with primary antibodies overnight at 4°C. After washing three times with PBS containing 0.1% Triton X-100, cells were incubated with Alexa 594-, 488- or 350-labeled anti-rabbit or anti-mouse IgG antibodies (Invitrogen) for 1 h. Cells were counterstained with DAPI and washed twice, then mounted in antifade agent on glass slides and visualized with an inverted fluorescence microscope (Olympus).

### Luciferase assay

Briefly, HEK293 or HeLa cells were seeded in 24-well plates and co-transfected with 100 ng of the appropriate Renilla luciferase reporter actin-DN or actin-LC3DN plasmids, 600 ng myc-RNF5 or shRNF5 expression plasmids, or with control plasmids. Transfections were performed using Lipofectamine 2000 (Invitrogen) according to the standard protocol. After 48 h, the medium was refreshed and then collected at different time points. The Renilla luciferase assays were carried out according to the manufacturer's instructions for the dual luciferase reporter assay system (Promega).

### Bacterial infection experiments

GAS strain NZ131 (serotype M49) was grown in Todd-Hewitt broth (THB, Difco, Detroit, MI) at 37°C to mid-logarithmic phase (OD600 = 0.4). For in vivo experiments, GAS were resuspended in PBS+5% mucin to an inoculum of 2×10^7^ CFU and injected intraperitoneally into WT or *RNF5^−/−^* mice. Three days post-infection, half of the mice were sacrificed and organs were collected and homogenized. Surviving bacteria in the organs were enumerated on Todd-Hewitt agar (THA) plates. The mortality of the remaining mice was monitored for an additional 7 days (10 days total). Mouse infection experiments were performed twice. For in vitro experiments, bone marrow cells were collected from age-matched WT and *RNF5^−/−^* mice and cultured in RPMI supplemented with 20% FBS and 30% L-929 cell conditioned medium for 7 days. Adherent macrophages were collected and seeded at 4×10^5^ cells per well in RPMI supplemented with 10% FBS and 10 ng/mL mouse macrophage colony-stimulating factor (Pepro-Tech, Rocky Hill, NJ) in 24-well plates for 1 day prior to bacterial infection or transfection with shRNA. Bone marrow-derived macrophages were then transfected with scrambled ATG4B or ATG5 shRNAs and bacterial infection was assessed 4 days later, as previously described [9], [58]. Intracellular survival was calculated as the percentage of bacteria remaining after antibiotics were removed from the culture medium.

To analyze the number of LC3 puncta in macrophages incubated with GAS, BM-derived macrophages were seeded on glass coverslips (5×10^4^/well in a 24-well plate) and infected on the following day with GAS expressing GFP (MOI = 10) for 3.5 h before washing and fixation in 3% paraformaldehyde (PFA). Cells were processed for immunofluorescence microscopy using affinity-purified rabbit anti-LC3 IgG, followed by goat anti-rabbit Alexa-488 F(ab′)2 and DAPI to stain the bacterial and cellular DNA. Images were taken on an Olympus TH4–100 fluorescence microscope using a 60× oil immersion lens (1.42 NA). Five Z-planes per field were captured and LC3 was quantified using Slidebook v.4.1 software.

### Electron microscopy

BM-derived macrophages were infected with GAS (MOI = 10) for 2.5 h. Cells were subsequently fixed with 4% PFA, 1.5% glutaraldehyde, and 5% sucrose in 0.1 M cacodylate buffer for 2 h at room temperature, postfixed in 1% OsO4 in 0.1 M cacodylate buffer for 1 h at room temperature, and then embedded as pelleted cells in LX-112 (Ladd Research, Williston, VT) as described previously [59]. Sections were stained in uranyl acetate and lead citrate and observed with an electron microscope (JEOL 1200 EX-II).

### Experiments in *C. elegans*

#### Strains

*C. elegans* strains were grown at 20°C according to standard protocols. The following integrated transgenes were used: [P_lgg*-1*_ *::gfp::lgg-1;rol-6*] [60], [61], [P_lgg*-1*_ *::gfp::lgg-1*;*rol-6*];*rnf-5(tm794)III* [62] (this study), [P_lgg*-1*_ *::gfp::lgg-1;rol-6*];*Ex*[*hsp-16p*::RNF-5;*P_myo-2_*::GFP] (this study), and *zcIs4*[*hsp-4p*::GFP] [47].

#### RNA interference

The complete *rnf-5*cDNA (708 bp) was cloned into the L4440 feeding vector (pPD129.36; a gift from Dr. Andrew Fire). RNAi by feeding was carried out as described previously [63]. Briefly, an overnight culture of the bacteria was inoculated 1∶100 and grown for 6 h at 37°C. The bacteria were concentrated 2-fold before seeding onto NGM-RNAi plates containing 1 mM isopropyl-d-thiogalactoside (IPTG; Sigma-Aldrich, St. Louis, MO) and 25 µg/ml carbenicillin. Plates were dried at room temperature for 2 days. Bacteria were induced with an additional 1 mM IPTG before adding embryos (P0). L4 larvae were transferred to freshly induced plates, and their progeny (F1) were analyzed at the L3 stage.

#### Microscopy and quantification

L3 larvae were mounted on 2% agarose pads and anesthetized with 0.1% tricaine and 0.01% tetramisole. Confocal analysis of GFP::LGG-1 puncta was performed using an LSM 5 EXCITER confocal scanning microscope (Carl Zeiss, Jena, Germany) by using a x63/1.4NA objective lens. GFP::LGG-1 puncta were counted using ImageJ analyze particles option, and significant differences between experimental groups were determined by two-tailed unpaired t-test, using SPSS.

#### RNF-5 overexpression

Transgenic worms expressing *rnf-5*cDNA under the *hsp16-2* and *hsp16-41* promoters [32] were grown at 25°C.

## Supporting Information

Figure S1 Human cDNA library screening for Atg4B inhibitors using a yeast-based protease activity assay. Human HepG2 cDNA library was transformed into yeast strain EGY48 harboring plasmids encoding ATG4B and the ATG4B-cleavable transcription factor substrate (pΔTEF3-ATG4B-FLAG-Gal1-Fas-d-LC3B-TA) and lacZ reporter gene (pSH18–34), then grown on selection plates for 4 days. Note that the cDNA library clones supported lacZ reporter gene activation only when co-expressed with WT ATG4B (but not with the cysteine/alanine active site mutant [C/A]) and only with the LC3-cleavable substrate (LC3) but not when using an alternative substrate containing a caspase-1 cleavage site (WEHD). From 2×10^5^ colonies, 12 hits appeared to inhibit Atg4B activity as monitored by lacZ reporter gene activity (β-galactosidase). Hit clone #11 was determined to contain a cDNA encoding full-length RNF5.
(EPS)

Figure S2 RNF5 interacts with, and affects, ATG4B. (A) ATG4B interacts with RNF5. Flag-ATG4B WT and C74A constructs and GFP-RNF5 expressing plasmids were co-transfected into 293T cells as indicated for 48 h. The cell lysates were immunoprecipitated with anti-Flag and the IP samples were detected with anti-GFP and anti-Flag antibodies as well as the input as indicated. (B) Dynamic interaction of ATG4B and RNF5 under starvation-induced autophagy. Ectopically expressed Flag-ATG4B and Myc-RNF5 plasmids were monitored for their interaction at the indicated times prior to and following starvation (HBSS), using IP and immunoblots with indicated antibodies. (C) ROS reduce ATG4B–RNF5 interaction. DTT, GSH, or H_2_O_2_ were added to the medium and incubated for 1 h. Then Flag-ATG4B was immunoprecipitated and the RNF5 interaction was analyzed. (D) RNF5 affects ATG4B–LC3 interaction. GFP-LC3 expressing plasmid was co-transfected into 293T cells in presence of shRNF5 or myc-RNF5 expressing construct, as indicated. Cell lysates prepared 48 h later were subjected to immunoprecipitation with anti-GFP antibodies and detected in immunoblots with anti-GFP and anti-ATG4B antibodies as indicated. SC, scramble shRNA.
(EPS)

Figure S3 RNF5 primarily interacts with and affects ATG4B. (A) Interaction between RNF5 and ATG4 isoforms. HA-tagged ATG4A-D isoforms and Flag-RNF5 constructs were co-transfected into 293T cells as indicated. The cell lysates prepared 48 h later were immunoprecipitated with anti-Flag antibodies and immunoblots were performed using anti-HA and anti-Flag antibodies. (B) Analysis of the interaction between endogenous ATG3, ATG5 and ATG7and ectopically expressed RNF5. HeLa cells stably expressing Flag-RNF5 were used for immunoprecipitation using Flag antibodies, followed by immunoblotting with the indicated ATG antibodies. (C) Expression of ATG3, ATG5, and ATG7 in RNF5 WT and KO MEF cells. Protein extracts prepared from WT and KO RNF5 MEFs were used for immunoblot analysis with the aid of the indicated ATG antibodies. Actin was used as a loading control. (D) Mapping the binding domains for ATG4B interaction with RNF5. 3Flag-ATG4B constructs were transfected into 293T cells with myc-RNF5 construct, 36 h later, the cell lysates were subjected to immunoprecipitation with anti-Flag M2 affinity gel. The IP complexes and inputs were detected as indicated. This result shows that 61–126 and 320–393 regions of ATG4B may be required for its interaction with RNF5. (E). Mapping the binding domains for RNF5 interaction with ATG4B. HA-ATG4B construct was transfected into 293T cells with a series of Flag-RNF5 constructs, which were subjected to 10 aa deletions within aa 61–180. Forty-eight hours later, the cell lysates were subjected to immunoprecipitation with anti-Flag M2 affinity gel and following with immunoblotting as indicated.
(EPS)

Figure S4 RNF5 mediates proteasome-dependent ATG4B degradation. (A) MG132 ablatesATG4B degradation in the presence ofRNF5 overexpression. Flag-RNF5 was transfected into 293T cells, and 24 h later, 10 µM MG132 was added for 4 h. (B) MG132 blocks ATG4B degradation by RNF5 in membrane fraction. The whole lysates and membrane fractions were prepared from RNF5 WT and KO MEF cells in the absence or presence of 10 µM MG132 (4 h), and analyzed by western blots as indicated. (C) Ubiquitination of endogenous ATG4B in membrane fractions from RNF5 WT and KO MEFs. The cells were incubated with or without MG132 (10 µM, 2 h), and protein extracts were subjected to sucrose gradient ultracentrifugation to enrich for organelles. The ubiquitination of ATG4B was analyzed with the indicated antibodies.
(EPS)

Figure S5 RNF5 reduces ATG4B activity. (A) ATG4B activity in vitro is attenuated by RNF5. Cell extracts were prepared from scrambled or shRNF5-transducedHeLa cells and maintained under normal growth conditions or subjected to HBSS (2 h) or DTT (5 mM, 8 h) treatment. ATG4B was immunoprecipitated and a fluorogenic-based assay was used to quantify ATG4B cleavage of in vitro synthesized pro-LC3 substrate. (B) LC3 cleavage in vivo is inhibited by RNF5. Actin-LC3DN (the reporter plasmid for LC3 cleavage) and Actin-DN (control plasmid) were co-transfected with shRNF5 expressing plasmids in HeLa cells. After 36 h, the medium was changed and incubated for an additional 4 h. The activities of Renilla luciferase were measured. Data shown are the mean of three experiments.
(EPS)

Figure S6 Disruption of RNF5 increases autophagy. (A) Supplementary to Figure 2A. LC3B, GABARAP1, and NBR1 were analyzed in RNF5 WT and KO MEFs during HBSS starvation. (B) RNF5 WT and KO MEF cells were grown under normal conditions or starved (HBSS, 2 h) and treated with E64D/PrestainA (2 h). LC3 were analyzed by western blotts. (C) RNF5 ΔCT fails to reduce level of LC3 puncta in RNF5 KO MEF cells. 2Flag-RNF5 WT and ΔCT constructs were transfected to RNF5 KO MEF cells. After 24 h, the cells were fixed and visualized with anti-Flag M2 and anti-LC3 antibodies. (D) Immunoblot depicting the expression of 2Flag-RNF5 WT and ΔCT constructs.
(TIF)

Figure S7 ATG4B is required for autophagosome formation. (A) Immunofluorescence images of mRFP-GFP-LC3 in ATG4B WT and KO MEF cells. The plasmid expressing WT or pre-cleaved mRFP-GFP-LC3 TFG was transfected into ATG4B WT and KO MEF cells. Twenty-four hours later the cells were fixed and visualized by microscopy. (B) Immunofluorescence images of mRFP-GFP-LC3 in RNF5 WT and KO MEF cells under starvation conditions. Supplementary to Figure 3B. The plasmid ptfLC3 expressing LC3 WT and pre-cleaved LC3 TFG were transfected into RNF5 WT and KO MEF cells. Twenty-four hours later the cells were starved with HBSS for 1.5 h, and then fixed and visualized by microscope.
(TIF)

Figure S8 Inhibition of RNF5 expression does not induce expression of hsp-4::GFP in *C elegans*, marker for ER stress. Basal expression of the *hsp-4::gfp* transcriptional reporter in *rnf-5(RNAi)*-treated worms. The *hsp-4::gfp* transgenic worms were fed with control (vector) or *rnf-5(RNAi)* bacteria in regular medium at 20°C.
(EPS)
