# Correction: Regulation of ATG4B Stability by RNF5 Limits Basal Levels of Autophagy and Influences Susceptibility to Bacterial Infection

**DOI:** 10.1371/journal.pgen.1008795

**Published:** 2020-05-11

**Authors:** Ersheng Kuang, Cheryl Y. M. Okumura, Sharon Sheffy-Levin, Tal Varsano, Vincent Chih-Wen Shu, Jianfei Qi, Ingrid R. Niesman, Huei-Jiun Yang, Carlos López-Otín, Wei Yuan Yang, John C. Reed, Limor Broday, Victor Nizet, Ze'ev A. Ronai

After this article [1] was published, concerns were raised about similarities between two pairs of panels in Fig 4:

- In Fig 4A, the first and second panels in the left column (GFP::LGG-1) appear similar.
- In Fig 4B, the first and fourth panels in the right column (*rnf-5(RNAi)*) appear similar.

**Fig 4:**
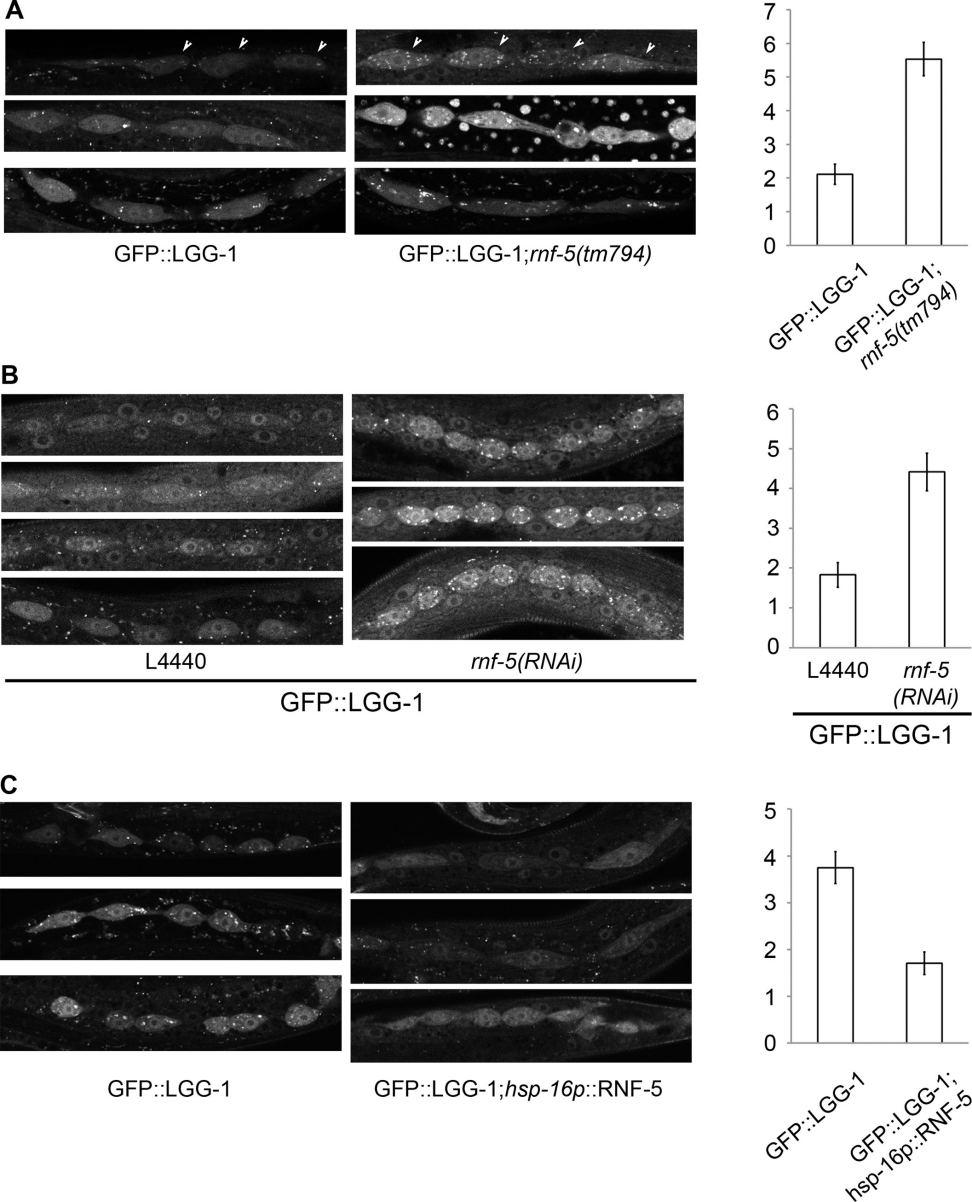
RNF-5 regulates autophagy in *C*. *elegans*. (A–C) Representative images of seam cells from L3 larvae expressing the GFP::LGG-1 transgene, and the average number of GFP::LGG-1 puncta in seam cells. Three to four independent experiments were performed for each condition. Error bars are ± SEM. P value was calculated using an unpaired two-tailed t-test. (A) *rnf-5(tm794)* larvae had an average of 5.53±0.49 puncta/cell compared to 2.11±0.30 puncta/cell in WT (36 cells from 9 *rnf-5(tm794)* larvae, and 46 cells from 10 WT larvae, p<0.0001). (B) *rnf-5(RNAi)*-treated worms had an average of 4.42±0.47 puncta/cell compared to 1.83±0.31 puncta/cell in control animals (77 cells from 15 *rnf-5(RNAi)* larvae and 64 cells from 17 control larvae, p<0.0001). (C) Animals grown constantly at 25°C: *hsp-16p*::*rnf-5* larvae had an average of 1.71±0.24 puncta/cell compared to 3.75±0.34 puncta/cell in the non-transgenic population (69 cells from 18 *hsp-16p*::*rnf-5* larvae, and 68 cells from 15 non-transgenic larvae, p<0.0001).

The authors noted that these panels were duplicated due to errors in figure preparation. An updated version of Fig 4 is provided here in which the duplicates are removed. The original image files supporting the updated figure are available upon request from the corresponding author.

The following images have also been removed in the updated figure: the second panel in right column of Fig 4A, the fourth panel in left column of Fig 4B, the third panel in left column of Fig 4C, and the fourth panel in right column of Fig 4C. These panels were correctly reported in the original figure but were removed in the revised version in order to balance the presentation of control and experimental images.

As noted in the figure legend, the images shown in Fig 4 are representative examples of worms harboring the reported changes. The authors confirm that the quantitative results shown in Fig 4A and 4B are unaffected by the image duplication errors. The quantitative data underlying the graphs in Fig 4A and 4C are provided in S1 and S2 Files. The raw quantitative data supporting Fig 4B are not available, for which the authors sincerely apologize. Notably, a set of independent experiments reveal the same pattern shown for Fig 4B (S3 File).

Raw data underlying all other results reported in the article are available.

The authors apologize for the errors in the published article.

## Supporting information

S1 File Original quantitative data underlying graph in Fig 4A. (XLSX)

S2 File Original quantitative data underlying graph in Fig 4C. (XLSX)

S3 File Replicate data supporting results in Fig 4B. (XLSX)
