# Supplementary figures and images for: Regulation of ATG4B Stability by RNF5 Limits Basal Levels of Autophagy and Influences Susceptibility to Bacterial Infection
Source: PLoS Genet. 2012 Oct 18;8(10):e1003007. doi: 10.1371/journal.pgen.1003007 (PMC3475677; doi:10.1371/journal.pgen.1003007)

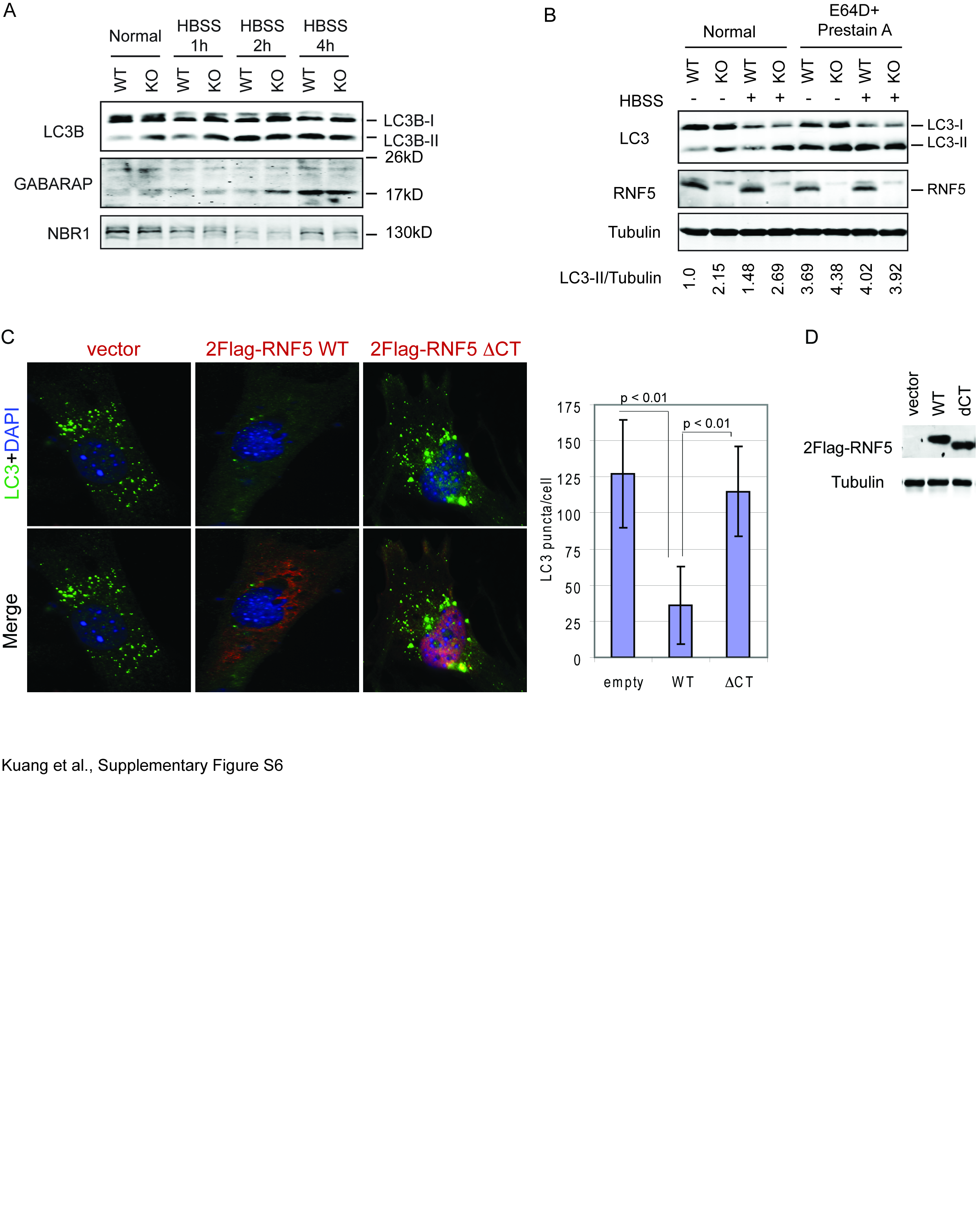

Supplement: Figure S6 — Disruption of RNF5 increases autophagy. (A) Supplementary to Figure 2A. LC3B, GABARAP1, and NBR1 were analyzed in RNF5 WT and KO MEFs during HBSS starvation. (B) RNF5 WT and KO MEF cells were grown under normal conditions or starved (HBSS, 2 h) and treated with E64D/PrestainA (2 h). LC3 were analyzed by western blotts. (C) RNF5 ΔCT fails to reduce level of LC3 puncta in RNF5 KO MEF cells. 2Flag-RNF5 WT and ΔCT constructs were transfected to RNF5 KO MEF cells. After 24 h, the cells were fixed and visualized with anti-Flag M2 and anti-LC3 antibodies. (D) Immunoblot depicting the expression of 2Flag-RNF5 WT and ΔCT constructs. (TIF) [file pgen.1003007.s006.tif]

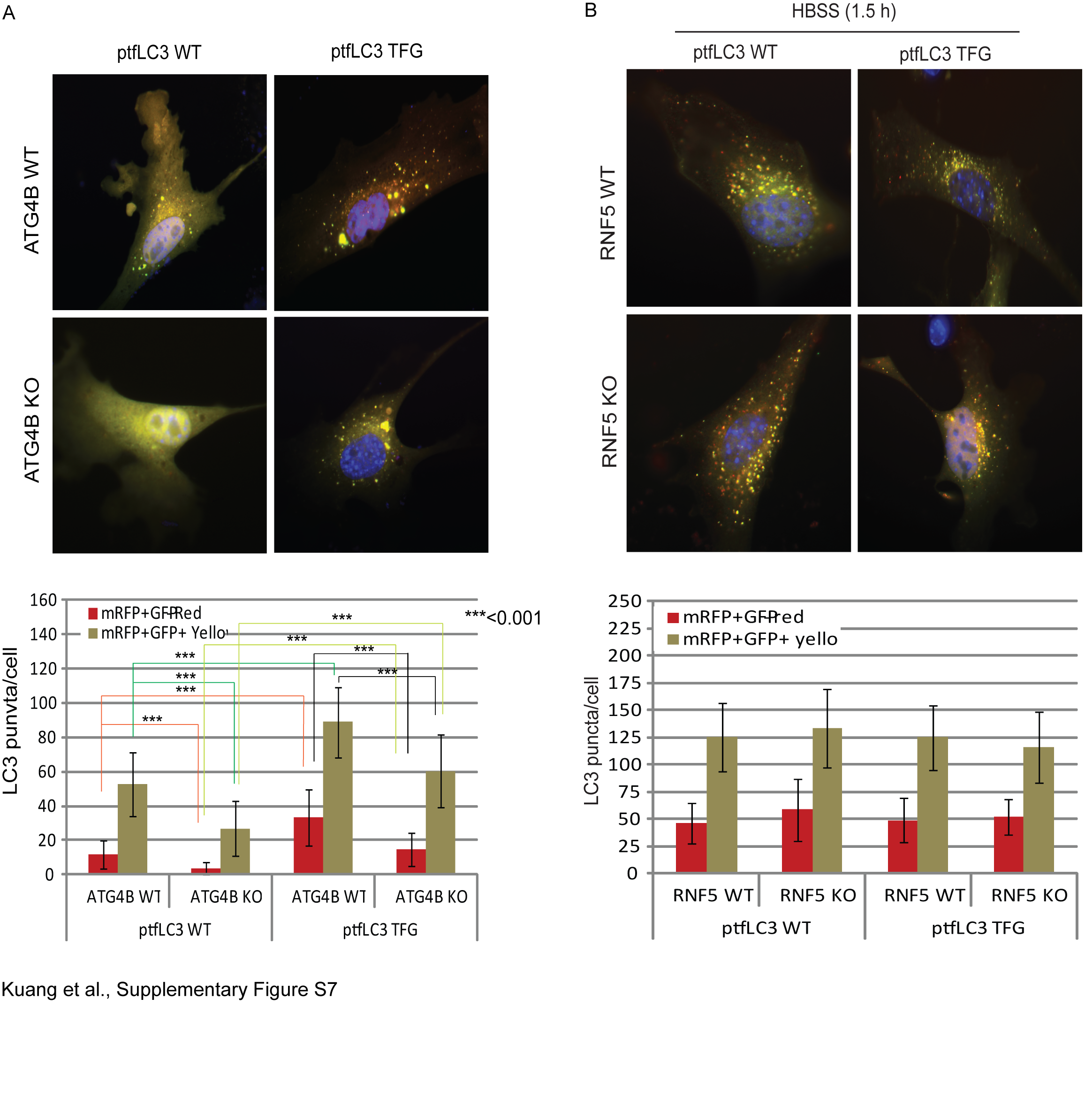

Supplement: Figure S7 — ATG4B is required for autophagosome formation. (A) Immunofluorescence images of mRFP-GFP-LC3 in ATG4B WT and KO MEF cells. The plasmid expressing WT or pre-cleaved mRFP-GFP-LC3 TFG was transfected into ATG4B WT and KO MEF cells. Twenty-four hours later the cells were fixed and visualized by microscopy. (B) Immunofluorescence images of mRFP-GFP-LC3 in RNF5 WT and KO MEF cells under starvation conditions. Supplementary to Figure 3B. The plasmid ptfLC3 expressing LC3 WT and pre-cleaved LC3 TFG were transfected into RNF5 WT and KO MEF cells. Twenty-four hours later the cells were starved with HBSS for 1.5 h, and then fixed and visualized by microscope. (TIF) [file pgen.1003007.s007.tif]
